# Lithium toxicity with prolonged neurologic sequelae following sleeve gastrectomy: A case report and review of literature

**DOI:** 10.1097/MD.0000000000021122

**Published:** 2020-07-10

**Authors:** Yi-Hsin Lin, Sheng-Wen Liu, Hsein-Lin Wu, Jung-Cheng Kang, Kuo-Yang Huang, Hsuan Huang

**Affiliations:** a Division of Endocrinology and Metabolism, Department of Internal Medicine; b Department of Internal Medicine; c Division of Pulmonology, Department of Internal Medicine; d Division of Proctology, Department of Surgery; e Department of Psychiatry, Taiwan Adventist Hospital; f Division of Pediatric Surgery, Department of Surgery, Mackay Memorial Hospital, Taipei, Taiwan (R.O.C.).

**Keywords:** bariatric surgery, lithium toxicity, neurologic sequelae, sleeve gastrectomy

## Abstract

**Rationale:**

Lithium is the first-line medication for bipolar disorder, given a narrow therapeutic window of 0.8 to 1.2 mEq/L. Change of lithium pharmacokinetics following bariatric surgery may lead to lithium toxicity, which is particularly concerned.

**Patient concerns:**

We presented a 39-year-old man with morbid obesity and bipolar affective disorder for 20 years, who was treated with lithium. He developed serious lithium toxicity following sleeve gastrectomy and prolonged neurologic sequelae.

**Diagnoses:**

He suffered from persistent watery diarrhea, general weakness, and then drowsy consciousness. Lithium level was checked immediately to be 3.42 mEq/L and lithium toxicity was diagnosed.

**Interventions:**

After 3 courses of hemodialysis, his serum lithium level subsequently declined to 0.63 mEq/L, while his consciousness returned normal. Lithium was replaced by lamotrigine.

**Outcomes:**

The patient was discharged thirty-five days after admission, while his serum lithium declined to 0.06 mEq/L. Neurologic sequelae were noted by muscle weakness and pain sensation in both feet. The nerve conduction test revealed sensorimotor polyneuropathy with conduction block. He was advised to keep a passive range of motion exercise.

**Lessons:**

Although the consensus guideline remains lacking, our report reviewed cases of relevance in the literature and highlighted the awareness of the potential risk of lithium toxicity following bariatric surgery. We suggest close monitoring of the lithium levels and perhaps a dosage adjustment for the postoperative period.

## 1 Introduction

Patients with bipolar disorder are more frequently associated with binge eating and obesity than the general population. McElroy et al reported that 14.3% of patients with bipolar disorder had at least one-lifetime comorbid eating disorder, in which binge eating disorder was the most common (8.8%) and associated with obesity and severe obesity.^[1]^ Nowadays, bariatric surgery is the most effective treatment for morbid obesity to reduce obesity-related comorbidity and total mortality.^[2]^ Vertical sleeve gastrectomy (SG) reduces substantial gastric volume by resection of a large portion of the stomach, and then patients achieve body weight loss. SG, accounting for 58% of bariatric surgery performed in the United States in 2016, has surpassed Roux-en-Y gastric bypass (RYGB) to become the most popular choice among all bariatric procedures.^[3,4]^ Notedly, around two-thirds of patients presenting for bariatric surgery received a psychiatric diagnosis, the most common of which was major depressive disorder.^[5]^ More than one-third of them took some psychiatric medication.^[5,6]^

Lithium is an effective mood stabilizer that is used as the first-line medication for bipolar disorder, given a narrow therapeutic window of 0.8 to 1.2 mEq/L.^[7,8]^ A study in Canada disclosed that the prevalence of lithium medication among patients undergoing assessment for bariatric surgery was around 1.2%.^[9]^ Change of lithium pharmacokinetics following bariatric surgery may lead to lithium toxicity, which is particularly concerned. We presented a case of serious lithium toxicity following SG and prolonged neurologic sequelae. Our report highlighted the awareness of the potential risk of lithium toxicity following bariatric surgery. We suggest close monitoring of the lithium levels and perhaps a dosage adjustment for the postoperative period.

### 1.1 Consent for publication

Written informed consent was obtained from the patient for publication of this case report and accompanying images. This case report was conducted under the Declaration of Helsinki. It was approved by the Institutional Review Board of Taiwan Adventist Hospital.

## 2 Case report

A 39-year-old male patient (173 cm, 135 kgm, body mass index: 45.1) was a case of morbid obesity. He had undergone laparoscopic SG for morbid obesity on August 21, 2018, which led to his poor appetite, nausea and vomiting. In following 3 weeks, he lost 17 kilograms of body weight. On September 16, 2018, he was sent to our hospital due to persistent watery diarrhea, dehydration, and general weakness for 3 days. Upon the arrival at our hospital, his vital signs were blood pressure 117/57 mm Hg, pulse rate 108/min, respiratory rate 18/min, body temperature 37.1°C. Electrocardiography showed sinus tachycardia. The laboratory data revealed severe hypokalemia (K 2.6 mmol/L [3.6–5.1]), acute kidney injury (Bun 29 mg/dL [8–20], Cr 4.36 mg/dL [0.44–1.03] estimated Glomerular filtration rate 16 [mL/min]), and normal liver, pancreas, cardiac enzymes as well as other electrolytes (Na 137 mmol/L [136–144], Cl 111 mmol/L [100–111], bicarbonate 26 mmol/L [22–26]) (Table 1). Other positive findings were mild anemia and leukocytosis (white blood cell 10.1^10^3^/uL [3.8–10.0], Hemoglobin 10.6 g/dL [12–16]), elevated C-Reactive protein 2.3 mg/dL (<1.0) and lactic acid 3.1 mmol/L (0.5–2.2).

**Table 1:**
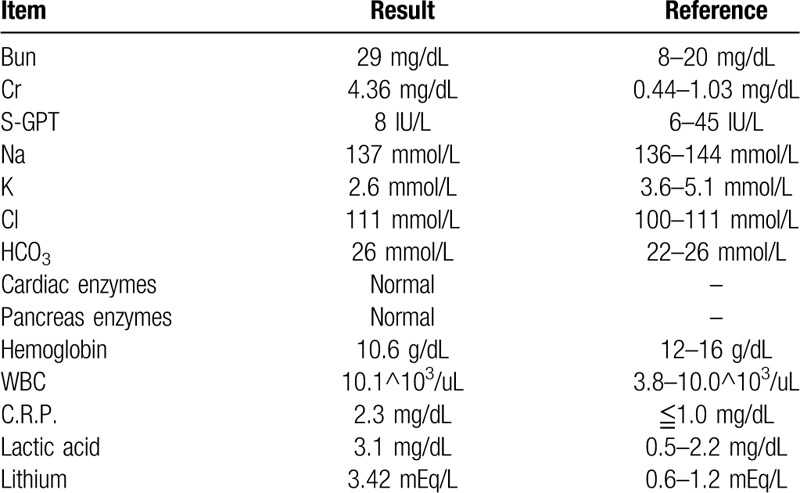
Initial laboratory data at initial presentation in September 2018.

He was admitted for aggressive intravenous hydration. On the third day of admission, the patient's potassium level (3.2 mmol/L) and kidney function (Bun 23 mg/dL, Cr 2.57 mg/dL, eGFR 30 mL/min) were getting better, but he was getting deteriorated with drowsy consciousness. The brain computed tomography revealed no significant findings. Tracing his medical history, he was diagnosed as a bipolar affective disorder for 20 years. He was under regular follow-up in the psychiatric clinic with lithium carbonate 600 mg twice daily, risperidone 2 mg twice daily, and flurazepam 60 mg once daily. He was still taking the same dosage during these days after surgery. Lithium level was found to be 3.42 mEq/L and lithium toxicity was diagnosed. Lithium carbonate was discontinued immediately, and urgent hemodialysis was suggested by nephrologist.

After 3 courses of hemodialysis, his serum lithium level subsequently declined to 0.63 mEq/L and creatinine level to 1.58 mg/dL, while his consciousness returned normal. In the following days, neurologic sequelae were noted by muscle weakness (muscle power 2 points over bilateral dorsi-flexors and plantar-flexors) and pain sensation in both feet. The nerve conduction test revealed sensorimotor polyneuropathy with conduction block. The patient was discharged thirty-five days after admission, while his serum lithium and creatinine level declined to 0.06 mEq/L and 1.05 mg/dl, respectively. He was advised to keep a passive range of motion exercise until the nerve function recovers. Lithium carbonate was replaced by lamotrigine now.

## 3 Discussion

We found 6 case reports of relevance in the literature.^[10–15]^ Four cases were presented after RYGB surgery and 2 cases after SG (Table 2). While several cases of lithium toxicity after RYGB were previously reported, reports after SG were still limited. In our report, we presented a case of serious lithium toxicity following SG and prolonged neurologic sequelae. The underlying causes of our patient's lithium toxicity are multifactorial.

**Table 2:**
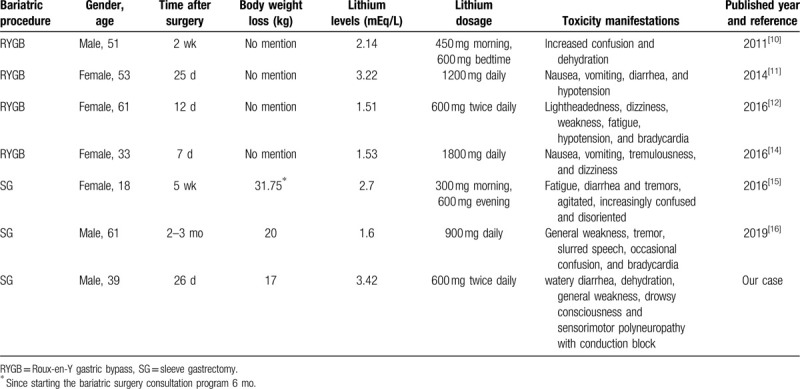
Literature review of lithium toxicity after bariatric surgery.

The modified gastrointestinal anatomy after bariatric surgery leads to significant alterations of pharmacokinetics in the oral absorption of drugs. The possible mechanisms, which are reviewed by Azran C, et al, are impaired gastric motility, decreased gastric volume and its acidity, reduced gastrointestinal transit time.^[16]^ These affect the drug dissolution, which is the rate-limiting step to drug absorption and has a significant influence on serum drug levels. In a previous in vitro study, investigators found that the dissolution of lithium was significantly increased in the post-RYGB surgery model.^[17]^ However, there is a lack of studies focused on the change in lithium absorption following SG. Since SG is presently the most frequently performed among bariatric procedures, further researches regarding its effects on lithium pharmacokinetics are needed.

Lithium is excreted nearly entirely from the kidney. Dehydration and acute kidney injury would lead to the decrement of serum lithium clearance and hence lithium intoxication.^[15,18]^ Due to gastric volume reduction after SG, our patient suffered from chronic gastrointestinal symptoms and was unable to intake adequate fluid, which led to a chronic dehydration status. Finally, he also manifested acute, persistent, watery diarrhea, which was a common symptom of lithium toxicity.^[19]^ The chronic dehydration status was further exacerbated by the severe volume depletion and led to impairment of kidney function, forming a vicious cycle that aggravated lithium toxicity in our patient. Moreover, 1 study revealed that the clearance of lithium was significantly greater in the obese population.^[20]^ The rapid weight loss of 17 kg in our case may also lead to decreased lithium clearance and toxicity under the same lithium dosage.

On the third day of admission, our patient's kidney function was getting better under aggressive intravenous hydration. However, his consciousness was getting drowsy, which was one of the findings of lithium neurotoxicity. Neurologic symptoms usually develop late in acute lithium intoxication, because lithium takes the time to be absorbed and to penetrate the central and peripheral nervous system. In some cases, neurologic complications did not disappear despite the successful removal of lithium by hemodialysis.^[21]^ These prolonged neurologic sequelae consist of cerebellar dysfunction, extrapyramidal symptoms, brainstem dysfunction, dementia, nystagmus, choreoathetoid movements, myopathy, and blindness.^[21,22]^ Demyelination in the nerve system was proposed as the cause, which was consistent with the nerve conduction test report of our patient, sensorimotor polyneuropathy with conduction block. The prolonged neurologic sequelae could continue for months and, in rare cases, for years.^[21,23]^

On account of the narrow therapeutic window of lithium, as well as the dramatic changes in oral intake and fluid status following bariatric surgery, caution should be taken in managing patients who take lithium. A protocol has been proposed by a previous literature, the authors of which suggest checking lithium level weekly during the first 6 postoperative weeks, then every 2 weeks until 6 months postsurgery, and later monthly until 1-year postsurgery.^[13]^ Although the consensus guideline remains lacking, close monitoring of lithium levels and perhaps a dosage adjustment are necessary for the postoperative period.

## 4 Conclusion

Patients on lithium undergoing SG or other bariatric procedures should be informed of the potential risk of lithium intoxication and its symptoms. Surgeons and psychiatrists should be alert to the signs and symptoms of lithium intoxication, especially for gastrointestinal problems which resemble the side effects of bariatric surgery and therefore could be misleading. Finally, further studies on lithium pharmacokinetics following bariatric surgery are needed to determine the most appropriate protocol for patients who take lithium and will undergo bariatric surgery.

## Acknowledgments

The authors would like to thank all the people who participated in this study. The authors also are grateful to the hospital officials for providing support for this study.

## Author contributions

**Data curation:** Yi-Hsin Lin, Sheng-Wen Liu, Hsein-Lin Wu, Jung-Cheng Kang.

**Investigation:** Yi-Hsin Lin, Sheng-Wen Liu, Kuo-Yang Huang.

**Resources:** Hsein-Lin Wu.

**Supervision:** Jung-Cheng Kang, Kuo-Yang Huang, Hsuan Huang.

**Validation:** Hsuan Huang.

**Visualization:** Hsuan Huang.

**Writing – original draft:** Yi-Hsin Lin, Sheng-Wen Liu.

**Writing – review & editing:** Yi-Hsin Lin, Hsuan Huang.
